# All Roads Lead to Interferon-γ: From Known to Untraveled Pathways in Acquired Aplastic Anemia

**DOI:** 10.3390/medicina59122170

**Published:** 2023-12-14

**Authors:** Bianca Serio, Valentina Giudice, Carmine Selleri

**Affiliations:** 1 Department of Medicine, Surgery, and Dentistry “Scuola Medica Salernitana”, University of Salerno, 84081 Baronissi, Italy; bianca.serio@sangiovannieruggi.it (B.S.); cselleri@unisa.it (C.S.); 2 Hematology and Transplant Center, University Hospital “San Giovanni di Dio e Ruggi d’Aragona”, 84131 Salerno, Italy

**Keywords:** acquired aplastic anemia, bone marrow failure syndromes, interferon-γ

## Abstract

Bone marrow failure (BMF) syndromes are a heterogeneous group of benign hematological conditions with common clinical features including reduced bone marrow cellularity and peripheral blood cytopenias. Acquired aplastic anemia (AA) is caused by T helper(Th)1-mediated immune responses and cytotoxic CD8^+^ T cell-mediated autologous immune attacks against hematopoietic stem and progenitor cells (HSPCs). Interferon-γ (IFNγ), tumor necrosis factor-α, and Fas-ligand are historically linked to AA pathogenesis because they drive Th1 and cytotoxic T cell-mediated responses and can directly induce HSPC apoptosis and differentiation block. The use of omics technologies has amplified the amount of data at the single-cell level, and knowledge on AA, and new scenarios, have been opened on “old” point of view. In this review, we summarize the current state-of-art of the pathogenic role of IFNγ in AA from initial findings to novel evidence, such as the involvement of the HIF-1α pathway, and how this knowledge can be translated in clinical practice.

## 1. Introduction

A long way of discoveries has been walked since the first described case of aplastic anemia in 1888 by Paul Ehrlich [1]. To date, our knowledge on aplastic anemia has completely changed, and the old terminology is kept only for historical reasons, as we now prefer the term bone marrow failure (BMF) syndromes [2]. Like Monet, who, despite observing the same Japanese bridge for years, was always able to grasp new details, making each painting different and more complex compared to the previous one, we might have been complicating an apparent simple reality. Currently, BMF syndromes are considered a heterogeneous group of benign hematological conditions with common clinical features, including reduced bone marrow (BM) cellularity and peripheral blood cytopenia(s) [1,2,3]. Based on pathophysiology, marrow failure syndromes are divided into constitutional, iatrogenic, and immune-mediated disorders [1]. Constitutional genetic defects are a subgroup of BMF syndromes caused by germline or somatic mutations in genes involved in hemopoiesis, DNA repair, or telomere biology [2]. Iatrogenic BMF is subsequent to a direct damage to the hematopoietic stem and progenitor cell (HSPC) compartment by external harmful factors, such as radiation, chemicals, drugs, and chemotherapy [1]. Immune-mediated BMF syndromes comprise four entities: acquired aplastic anemia (AA); paroxysmal nocturnal hemoglobinuria (PNH); hypoplastic myelodysplastic syndromes (MDSs); and large granular lymphocyte leukemia (LGL) [4]. However, these syndromes share numerous clinical, biological, and molecular features, and often coexist. For example, AA patients can display a small PNH clone at diagnosis, or can develop myelodysplasia over their disease course in 10–20% of cases [5]. This clinical and biological overlap underlies common pathogenic mechanisms, mainly an immune response dysregulation, albeit with differences, and a similar risk to progress to acute myeloid leukemia (AML) [6]. BMF syndromes are also a continuum of clinical entities that fade into other clonal hematological conditions, because they can harbor somatic mutations in AML-related genes, such as *ASXL1* or *DNMT3A* [1,2,3,4,5,6].

In normal hemopoiesis, the immune system is fine-tuned; conversely, in immune-mediated BMF, cell subsets and cytokines are variously deranged, resulting in a wide range of clinical manifestations. Historically, T helper(Th)1-mediated immune responses and cytotoxic CD8^+^ T cell(CTL)-mediated autologous immune attacks against HSPCs are the main pathogenetic event in AA [1,2]. The normalization of blood counts after immunosuppression is the main indirect evidence of the immune-mediated origin of this entity [7]. One of the first and still the most supported pathogenetic hypothesis assumes that an unknown viral infection affecting stem cells could cause a cross-reactivity with self-antigens and the subsequent expansion of an autoimmune clone [4]. Infected cells preferentially activate Th1 cells, which are the predominant CD4^+^ T lymphocytes involved in viral clearance through the activation of CTLs via interferon-γ (IFNγ) or tumor necrosis factor-α (TNF-α) [6,8]. CTLs directly kill HSPCs through Fas-ligand (FasL) secretion [9]. However, based on new discoveries, other ancillary mechanisms contribute to AA pathogenesis and/or the consolidation of altered immune responses [6]. For example, Th17- and effector memory-mediated responses are predominant in subjects with long-lasting and severe disease, while Th1 and CTLs are predominant in new-onset AA [6,7,10].

## 2. Pathogenic Role of IFNγ in AA

IFNγ and FasL are historically linked to AA pathogenesis. First studies on the driving role of IFNγ in AA are dated back to 1995 [11]. In these first experiments, long-term stem cells (LTSCs) were cultured on an engineered marrow stroma, producing IFNγ and/or TNF-α, and showed a constant increasing apoptotic rate only when adhered to stromal cells [11]. Furthermore, IFNγ-treated LTSCs display a higher surface expression of FasL, an apoptotic inducer, thus promoting CTL-mediated cytotoxicity against HSPCs [9,12]. Moreover, IFNγ and, less markedly, TNF-α induce the expression of inducible nitric oxide synthase that promotes apoptosis through nitric oxide production [9,13]. When cultured on engineered IFNγ-producing stroma, HPSCs undergo apoptosis under a 100-times lower amount of IFNγ compared to those concentrations required in culture medium to obtain the same effect [11]. Therefore, IFNγ exerts its action in the near proximity of the cell surface and through lymphocyte-to-HSPC contact within the BM, thus justifying the observation of normal circulating levels in the majority of AA patients [14,15]. After this discovery, several studies have identified new cytokines, chemokines, growth factors, and other deregulated molecules that might play a central or ancillary role in AA pathophysiology [1]. For example, Th1-related response cytokines (e.g., interleukin (IL)-12 and IL-23) can amplify IFNγ production by T lymphocytes and Natural Killer (NK) cells and consolidate Th1 response [6]. Upon stimulation, Th1 lymphocytes secrete IL-2 and IFNγ and activate macrophages and CTLs. Therefore, a “biting-tail dog” inflammatory mechanism is triggered and auto-sustained. Moreover, this loop is maintained as long as HSPC-derived self-antigens are present and induce the autologous autoimmune attack, as IFNγ and TNF-α BM plasma levels are elevated at diagnosis and correlate with disease severity, while they decline after recovery [14,15]. However, circulating levels of these cytokines could not be increased, supporting the hypothesis that the autoimmune attack is confined to the BM [4]. Indeed, the eradication of granulocytic myeloid-derived suppressor cells, a tissue-specific antigen-presenting cell population with immunomodulatory functions, ameliorated BMF in a murine model of AA [16].

**Figure 1:**
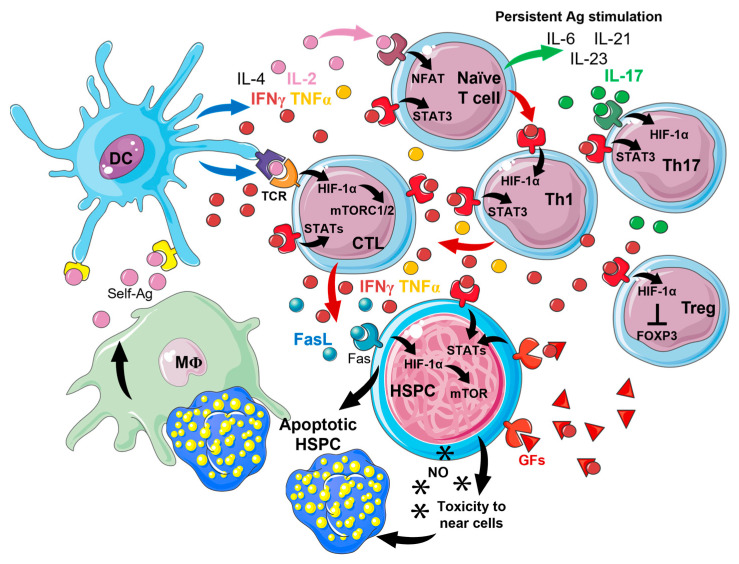
Pathophysiology of acquired aplastic anemia (AA). Apoptotic and/or infected hematopoietic stem and progenitor cells (HSPCs) can release unmodified or chemically and/or genetically modified antigens (self-Ags) that are phagocyted by macrophages (Mφs) and presented to antigen-presenting dendritic cells (DCs). These cells process and present Ags to naïve CD4^+^ T cells, that differentiate toward T helper (Th) 1 phenotype under interleukin (IL)-2, IL-4, tumor necrosis factor alpha (TNF-α), and interferon-gamma (IFNγ), mainly through STAT and HIF-1α signaling pathway activation. DCs via Ag presentation to T-cell receptor (TCR) and Th1 cells sustain effector cytotoxic CD8^+^ T lymphocyte (CTL) activation through STATs, HIF-1α, and mTORC1/2 signaling. Activated CTLs induce growth inhibition and apoptosis of HSPCs by directly killing cells via granzyme B and perforin release, and by paracrine inhibitory effects of TNF-α, IFNγ, Fas ligand (Fas-L), activation of inducible nitric oxide (NO, *) synthase, and NO release, they also exert cytotoxicity near cells. HSPC growth inhibition is also caused by a decoy receptor function of IFNγ, that binds to growth factors (GFs), such as thrombopoietin, and interferes with their binding to surface receptors. Persistent Ag stimulation induces Th17 differentiation of naïve T cells under IL-6, IL-21, and IL-23 stimulation. Th17 cells also reduce T regulatory cell (Treg) function and numbers because IL-17 and IFNγ augment HIF-1α stabilization which blocks the master transcription factor FOXP3 of Treg. Parts of the figure were drawn by using pictures from Servier Medical Art. Servier Medical Art by Servier is licensed under a Creative Commons Attribution 3.0 Unported License (https://creativecommons.org/licenses/by/3.0/) (accessed on 15 November 2023).

IFNγ and TNF-α also exert a direct inhibitory effect on HSPC growth and differentiation [17]. Exogenous and endogenous IFNγ (produced by stromal BM cells) can interfere with growth factor signaling pathways, especially the thrombopoietin (TPO)-TPO receptor (c-MPL) axis [18]. Under inflammatory conditions, IFNγ can bind to hematopoietic growth factors, acting as a decoy receptor and drastically reducing their activity and ability to interact with partner ligands on the HSPC surface. In non-responder (NR) AA patients, after immunosuppressive therapies, IFNγ remains augmented, thus justifying persistently high circulating TPO levels [19]. In this case, this growth factor is trapped by IFNγ and is non-functional, and megakaryopoiesis is not properly sustained; therefore, platelet counts remain low, and TPO is continually produced to over-ride this IFNγ-mediated block [18,19]. Moreover, IFNγ induces the expression of IL-15, a cytokine that blocks erythroid precursors at the earliest stages of differentiation, ultimately leading to reduced erythropoiesis [20]. T-bet^+^ cells also produce high amounts of IFNγ [21,22]. This T cell subset is essential for Th1 differentiation and IFNγ production during inflammation, and halts CD4^+^ T cell differentiation towards other Th subsets [23]. Indeed, T-bet expression is irreversibly associated with Th1 differentiation and is important for the terminal differentiation of memory CD4^+^ and CD8^+^ T cells during acute infections and for preventing CD8^+^ T cell exhaustion during chronic antigen stimulation [23,24] (Figure 1).

IFNγ is involved in several pathways, such as those mediated by cytokine stimulation; HIF-1α, TGF-β, JAK/STAT or other cytokines; those pathways involved in Th differentiation; antigen processing and presentation; T cell receptor signaling; NK-mediated cytotoxicity; T cell exhaustion and other pathways in cancers; autoimmune disorders; and infectious diseases; as reported in KEGG database (v.108; 2023/10) [25,26,27,28]. When these pathways were interpolated with those previously identified in CR and PR/NR, two common pathways were found: HIF-1α signaling and pathways in cancer. HIF-1α, an oxygen sensitive subunit of HIF-1, is expressed under hypoxic conditions and is also constitutively transcribed after growth factor and cytokine stimulation [29]. In normoxic conditions, HIF-1α is quickly degraded; however, growth factors, such as erythropoietin, can stabilize and enhance the transcriptional activity of HIF-1α under hypoxic environments [30].

Upon stimulation by interleukins, IFNγ, or other growth factors; phosphatidyl inositol-4,5-bisphosphate-3-kinase (PI3K); and MEK are activated, and signal transduced through protein kinase B (Akt) and the mammalian target of rapamycin (mTOR) that disrupts the eukaryotic translation initiation factor 4E (eIF-4E) binding protein (4E-BP1) and allows for the nuclear translocation of HIF-1α [29]. After translocation, this factor induces the transcription of multiple genes, such as VEGF and EPO, and other genes related to cell proliferation and survival, like Bcl-2 and p21/p27. However, under physiological hypoxic conditions, HIF-1α only plays a dispensable role in HSPC maintenance, as unstressed stem cells carrying the acute deletion of HIF-1α efficiently self-renew and differentiate upon serial transplantation [31]. Conversely, HIF-1α is essential for macrophage polarization, and T and B cell responses, especially upon IFNγ stimulation [32]. For example, during the mycobacterium tuberculosis (MTB) infection of macrophages, HIF-1α is induced and determines the secretion of pro-inflammatory cytokines, such as IL-1β [33]. On the other hand, during MTB infection, IFNγ is also produced and causes MTB growth restriction in macrophages, and binds to HIF-1α to produce lipid droplets via macrophages as part of the immune response against the mycobacterium [34]. IFNγ and HIF-1α play in concert during immune responses, as almost half of IFNγ-induced genes are also HIF-1α-dependent [35]. Moreover, HIF-1α is important in dendritic cell (DC) maturation and DC-mediated T cell activation, and in neutrophil survival and degranulation [36,37].

TNF-α acts as a companion of IFNγ during AA, as its BM levels and he expression of its receptors are increased [1,25]. Based on BMF mouse model evidence, macrophages are the major source of TNF-α in AA, and the principal targets are lymphocytes, as the TNF-α receptor gene knockdown results in reduced T cell migration and the secretion of IFNγ and TNF-α through the upregulation of the master transcription regulator Tbx21 [38].

## 3. Targeting IFNγ: A Double-Sided Coin

**Figure 2:**
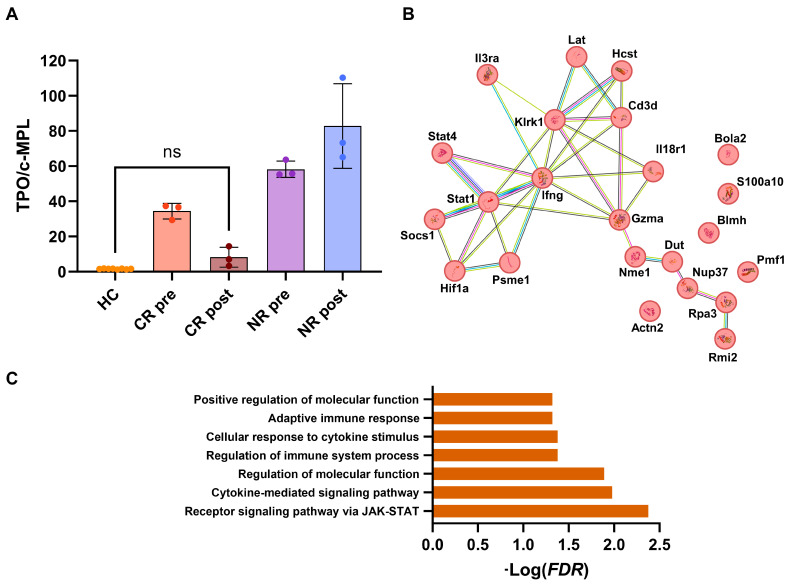
Thrombopoietin (TPO) axis and ruxolitinib-related changes in AA mouse model. (**A**) Circulating plasma levels of TPO and its receptor c-MPL from [28] were extracted. TPO/c-MPL ratio was calculated for each healthy control (HC), complete (CR), partial (PR), and non-responder (NR) group before (pre) and after (post) immunosuppressive therapy + eltrombopag, and levels compared by one-way ANOVA. All comparisons corrected with Tukey’s multiple comparisons test were statistically significant, except between HC and CR post-treatment (*p* = 0.8303). (**B**) Putative gene network interactions using dysregulated genes in CD8^+^ T cells from ruxolitinib-treated BMF mice from [51] were interpolated by also including HIF-1α, and (**C**) protein pathways were identified by STRING database. ns, not statistically significant.

Currently, AA patients not eligible for allogeneic hematopoietic stem cell transplantation are treated with cyclosporine A (CsA), anti-thymocyte globulin (ATG), and eltrombopag as the first-line treatment [2]. CsA and ATG are potent immunosuppressors, targeting deregulated immune responses in AA and inducing an immunological reset. CsA, a calcineurin inhibitor, blocks T cell activation by dephosphorylation of the nuclear factor of activated T cells (NFAT), a signaling transducer of the TCR and IL-2 pathways [6,39,40]. CsA also inhibits T lymphocyte proliferation by reducing IL-2, IL-3, and IFNγ expression [39,40,41,42]. ATG is a lymphodepleting agent and interferes with adhesion molecule and chemokine receptor expression, DC functions, and B and T cell survival by the promoting expansion of Treg and NK [43]. Eltrombopag, a TPO agonist, was first employed in AA as a stimulator of HSPC growth and differentiation based on its benefits in immune thrombocytopenia [41]. However, bi- or tri-lineage hematologic recovery has opened new questions regarding its mechanisms of action. Numerous studies have demonstrated the immunomodulatory activity of eltrombopag in AA, MDS, AML, and post-transplant poor graft function (PGF) [44]. This TPO agonist can increase Treg and B regulatory cell (Breg) frequency and restore immune tolerance via effector memory T cell reduction. Eltrombopag can also induce the secretion of immunosuppressive cytokines and halt pro-inflammatory molecule expression through JAK/STAT signaling pathway modulation and TET2 inhibition [45,46]. In addition to these immunomodulatory effects, eltrombopag increases HSPC survival and fitness through two principal mechanisms: iron chelation and oxidative stress reduction; and the removal of the IFNγ block [47,48,49]. Indeed, in AA, IFNγ is in excess and acts as a decoy receptor for TPO that cannot not bind to its receptor c-MPL and trigger signaling transduction, leading to HSPC proliferation and survival. Conversely, eltrombopag skips this block by binding c-MPL to a different site and can induce HSPC growth [49]. Several studies have demonstrated a persistent increase in circulating TPO levels in AA and also in responders in a long-term follow-up, even though circulating levels are lower than those observed in NR [19]. This finding still has unclear implications and explanations, also because circulating c-MPL levels are persistently reduced in AA patients regardless of responsiveness to immunosuppressive therapies, with or without eltrombopag [50]. We re-analyzed published data obtained from an aptamer-based proteomics analysis of plasma AA samples, and circulating levels of TPO and the c-MPL ratio were calculated because in normal conditions, free TPO should bind its natural ligand, c-MPL, on either cell surface or to the decoy circulating receptor. Indeed, in healthy controls, this ratio was very low (mean ± SD, 1.59 ± 0.2 RFU), while it was significantly augmented in AA subjects at diagnosis (mean ± SD, 50.6 ± 13.7 RFU) and remained elevated in NR after treatment (mean ± SD, 82.8 ± 24.1 RFU), while it was significantly decreased in CR (mean ± SD, 8.2 ± 5.7 RFU; healthy controls vs. CR, *p* = 0.8303) (Figure 2A). This evidence supports the hypothesis that TPO is trapped by IFNγ in the proximity of the HSPC surface, preventing its binding to surface c-MPL and signaling transduction. Eltrombopag can first directly induce HSPC growth through c-MPL activation and modulate immune response by reducing IFNγ production, subsequently restoring normal growth factor signaling transduction.

In a recent work, the efficacy of ruxolitinib, the first JAK1/2 inhibitor, in preventing AA onset in mouse models was investigated. Ruxolitinib-treated mice showed a reduced production of pro-inflammatory cytokines, as widely described in myelofibrosis, autoimmune disorders, and SARS-CoV-2-related cytokine release syndrome [51,52]. Treated mice showed increased survival, and their marrow does not exhibit typical aplasia, regardless of the timing of ruxolitinib administration (after four or six days of AA induction). Lymphocytes of ruxolitinib-treated mice are also “turned off”, and numerous pathways related to lymphocyte activation and cytotoxic activity are downregulated. Furthermore, downregulated genes in cytotoxic CD8^+^ T cells from ruxolitinib-treated mice include *IFNG*, *STATs*, and *SOCS1* [51]. If we re-analyzed these subsets of mouse proteins by including Hif1a (mouse ortholog of human HIF-1α) for protein pathway analysis using the STRING database (v.12.0), 22 proteins (52% of included proteins, n = 42) clustered with Hif1a. These proteins are involved in the following: receptor signaling pathway via JAK/STAT; cytokine-mediated signaling pathway; the regulation of molecular function and immune system process; cellular response to cytokine stimulus; adaptive immune response; and the positive regulation of molecular function. Therefore, the same proteins and pathways seemed to be related to AA pathogenesis, even in mouse models, and drugs targeting JAK/STAT signaling or interfering with IFNγ-HIF-1α axis have therapeutic effects in mitigating immune responses and inducing HSPC growth and differentiation.

## 4. New Perspectives on Proteins Involved in AA Pathogenesis

Omics technologies have amplified the amount of data at the single-cell level and knowledge on AA [8]. A large-scale aptamer-based proteomic serum analysis has identified more than 150 proteins associated with the clinical response to immunosuppressive therapies. Only four of them (C-C motif chemokine ligand 17 (CCL17), Dickkopf WNT signaling pathway inhibitor 1 (DKK1), hepatocyte growth factor (HGF), and L-selectin (SELL)) have been validated in a larger cohort of patients [50]. These validated biomarkers were chosen based on the availability of the multiplex assay and not on biological relevance [50]. In this study, signatures of responsiveness to immunosuppressive therapies were extrapolated using two-way ANOVA across groups before and after treatment or unpaired *t*-test with FDR correction (5%) between groups, and proteins that showed differences only after treatment were excluded. Using this strategy of analysis, only 19 proteins have been identified as a proteomic signature of responsiveness to immunosuppressive therapies, with other biomarkers likely having been missed because of this conservative analytical approach and the small sample size of the discovery cohort in reported studies [50]. For those selected proteins, several enriched networks have been described, suggesting that dysregulated molecules are not randomly increased or decreased in the sera of AA patients, which might have roles in disease pathophysiology. For example, several proteins are associated with the Wnt pathway, and their normalization is frequent in AA responders [50]. Wnt signaling is important in early hematopoietic ontogeny and the maintenance of self-renewing LTSCs after stress [53,54,55]. Conversely, the inhibition of Wnt by DKK1 impairs BM recovery after transplantation, reduces LTSCs, increases cell cycling, and promotes myeloid compartment expansion [55,56]. The regulation of Wnt is complex as multiple inhibitors and activators are involved [57]. From these aptamer-based signature proteins, four molecules are associated with Wnt signaling pathway: three are negative regulators (DKK1, DKK4, and FRZB), while WISP1, downstream of WNT1, is increased in many tumors and inflammatory diseases [58].

**Figure 3:**
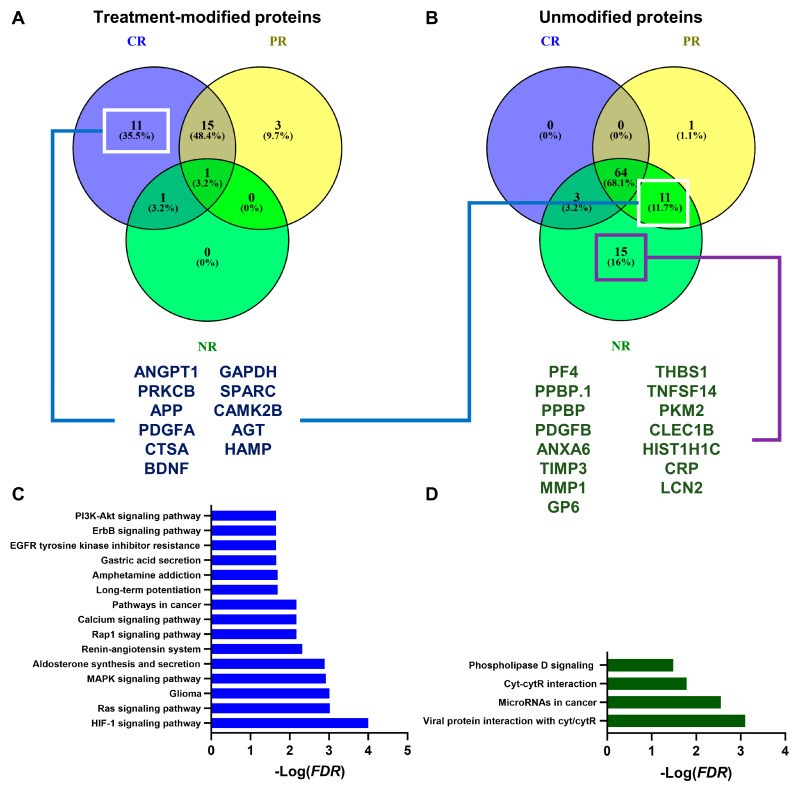
Protein pathway analysis in acquired aplastic anemia (AA). Proteins from an aptamer-based high-throughput proteomic analysis [28] were extracted, and those whose levels changed >1.5 fold change after immunosuppressive therapies in complete (CR), partial (PR), and non-responders (NRs) were interpolated. (**A**) Proteins that showed >1.5 fold changes (n = 11) and (**B**) that did not change after treatment in PR and NR (n = 11) nor in NR only (n = 15). (**C**) Those 11 proteins were used for protein pathway analysis by STRING database, and the first top 15 represented networks are reported, as well as (**D**) those networks enriched using those 15 unmodified proteins in NR only.

This large-scale proteomics analysis of AA sera and plasma is mainly an undiscovered mine; therefore, we can try to dig deeper into SOMAscan published data. Non-responders (NRs), complete (CR) or partial responders (PRs), and a group of healthy controls (n = 14; M/F, 6/8; mean age, 32.3 years old (range, 21–62)) are included in this dataset, and the results were compared before or after therapies by unpaired *t*-test (Supplementary Table V of [50]). More than 200 proteins were found to be increased or decreased in healthy subjects compared to AA sera, using a 1.5 fold-change expression as the cutoff, as previously described [50]. However, we tried to re-analyze those data, and proteins that vary more than 1.5 fold between healthy controls and CR, PR, or NR before treatment were identified. Subsequently, variations before and after treatment were assessed by subtracting fold-change values calculated between healthy subjects and each AA group after therapy to fold change values before treatment, as follows: protein level variation = fold change (Healthy vs. AA) after treatment—Fold change (Healthy vs. AA) before treatment. Using this formula, proteins that showed variations after treatment—even those normalized to control levels—could be identified as targets of immunosuppressive therapies. As expected, no proteins varied in NR after treatment, while 28 were modified by immunosuppressors in CR, and 15 of them were in common with PR (Figure 3).

Those 15 common proteins were the following: PF4, PPBP.1, PDGFB, ANXA6, TIMP3, MMP1, GP6, THBS1, CCL28, TNFSF14, PKM2, CLEC1B, HIST1H1C, CRP, and LCN2. Those 11 proteins present only in the CR group were: ANGPT1, PRKCB, APP, PDGFA, CTSA, BDNF, GAPDH, SPARC, CAMK2B, AGT, and HAMP (Figure 3A). Protein pathway analysis was performed using STRING database [59], and common proteins were related to the following: viral protein interaction with cytokine and cytokine receptor; microRNAs in cancer; cytokine–cytokine receptor interaction; and phospholipase D, Rap1, PI3K-Akt, JAK-STAT, chemokine, calcium, Ras, and MAPK signaling pathways; EGFR tyrosine kinase inhibitor resistance; and other cancer pathways (Figure 3C). Those proteins present only in CR were associated with the following signaling pathways: HIF-1, Ras, MAPK, Rap1, calcium, ErbB, or PI3K-Akt signaling; and other pathways in cancers. Protein levels that were not modified after treatment in PR and/or NR were identified as biomarkers of non-responsiveness to immunosuppressive therapies, and a total of 26 proteins were selected: ANGPT1, PRKCB, APP, PDGFA, CTSA, BDNF, GAPDH, SPARC, CAMK2B, AGT, and HAMP in both PR and NR; and PF4, PPBP.1, PPBP, PDGFB, ANXA6, TIMP3, MMP1, GP6, THBS1, TNFSF14, PKM2, CLEC1B, HIST1H1C, CRP, and LCN2 in NR only (Figure 3B). Interestingly, those 11 unmodified proteins after treatment in PR and NR were the same 11 molecules that were identified as protein signatures in CR after treatment. Conversely, those 15 unmodified proteins in NR were enriched in only four signaling pathways: viral protein interaction with cytokine and cytokine receptor; microRNAs in cancer; cytokine–cytokine receptor interaction; and phospholipase D signaling (Figure 3D). Protein pathways were also visualized using a different database, Reactome v.86 [60,61], showing a different distribution between CR and PR/NR of the involved pathways, especially immune responses, hemostasis, and signaling transduction.

## 5. IFNγ: From Old-Fashioned to New Molecules

HIF-1α is important in T cell differentiation in both normoxic and hypoxic environments with differences in each T cell subset, as T lymphocytes show various glycolytic activity, with Th17 being the most sensitive to glycolytic induction and T regulatory cells (Treg) being the least sensitive [62]. Indeed, HIF-1α is highly upregulated in Th17 cells, likely induced by IL-6, IL-21, and IL-23 stimulation; STAT3 activation; and by the master regulator of Th17 differentiation, RORγt, while this factor is expressed at low levels in Treg [63,64]. Under hypoxic conditions, STAT3 activation is augmented in Th1 cells, leading to HIF-1α and IFNγ increase; however, Th1 cells inhibit *IFNG* transcription, and the proportion of IFNγ-producing cells is decreased [64]. Conversely, under normoxic conditions, HIF-1α-induced *IFNG* transcription is not repressed, and IFNγ is upregulated, while FOXP3 is downregulated, resulting in Th1 increase, Treg decrease, and the establishment of a pro-inflammatory environment [65]. HIF-1α is also important in CD8^+^ cytotoxic T cell activation following TCR stimulation through mTORC1 and 2 transduction, and promotes effector and memory CD8^+^ T cell differentiation [66,67]. Moreover, CD8^+^ T cells increase the expression of IFNγ and TNFα upon TCR-dependent HIF-1α upregulation [68]. In AA, naïve T cells differentiate toward the Th1 phenotype following IFNγ, TNFα, and other type I IFN cytokine stimulation, and activate CD8^+^ cytotoxic T cells directed against HSPCs. The chronic release of self-antigens by HSPCs determines naïve T cell differentiation toward the Th17 phenotype, the terminal differentiation of cytotoxic T cells in effector memory CD8^+^ T lymphocytes, and Treg reduction [4,6]. These findings might be in accordance with hypothesized HIF-1α-induced effects on AA-related immunity.

HIF-1α involvement in AA pathogenesis can also be investigated in other BMF syndromes to support its role in impaired immune responses in these diseases based on their clinical and biological overlap. A recent study using single-cell TCR coupled with the RNA sequencing of CD3^+^ T cells from LGL patients has shown the deregulation of genes involved in cell survival and apoptosis. The marked downregulation of apoptosis genes in CD8^+^ neoplastic cells before treatment has been shown, while they are upregulated in responder patients after alemtuzumab [69]. Among those upregulated or downregulated gene networks, *HLA*, *IFNG*, *CD8A/B*, *CD38*, *STAT1/3/4*, *IL7*, *CCL3/4/5*, *IL21R*, *FAS* and *FASLG*, *SOCS1*, *TNFRSF1B*, *PRKCB*, *IL1B*, *BIRC3*, *TNF*, and *MTOR* genes are deregulated in LGL before treatment. Of those genes, *IFNG*, *STAT3*, *TNF*, *BIRC3*, *IL7R*, *SOCS1*, *TNFRSF10A*, *IL2RA*, *IL6ST*, *FASLG*, *GATA3*, and *AKT3* are downregulated after alemtuzumab [69]. Of note, *IFNG* gene is found to be deregulated at diagnosis and downregulated after therapy, confirming its driving role in BMF syndrome pathogenesis.

**Figure 4:**
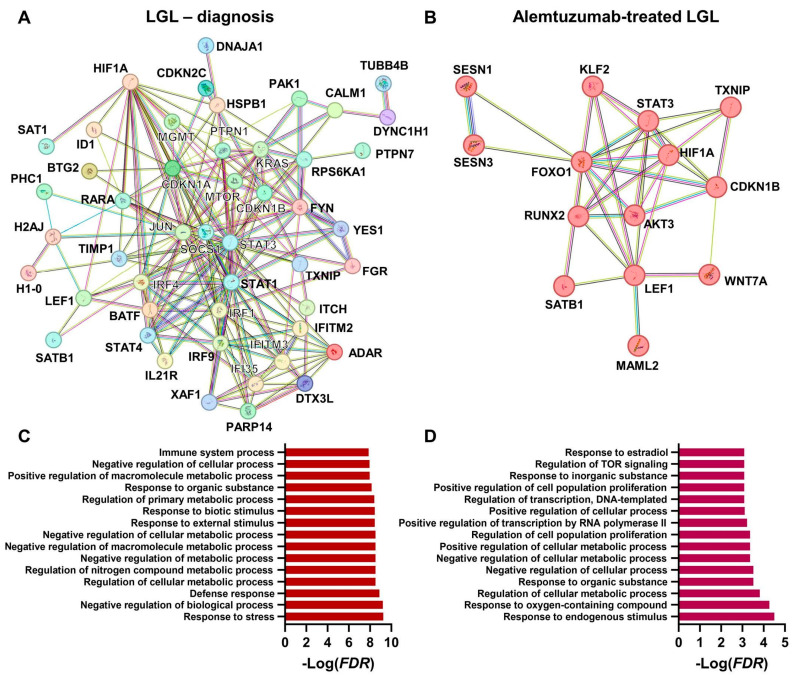
Protein pathway analysis in large granular lymphocytic leukemia (LGL). Genes from a single-cell TCR coupled with RNA sequencing of CD3+ T cells from LGL [65] were extracted, and those upregulated or downregulated (**A**) at diagnosis and (**B**) after alemtuzumab treatment were used for protein pathway analysis using the STRING database and by also adding HIF-1α. First 15 that better represented networks are reported for (**C**) diagnosis and (**D**) after treatment.

From another protein pathway analysis performed including HIF-1α in these two subsets of proteins, we showed that this factor could be implicated in disease pathogenesis and responsiveness to therapy. Using the STRING database, proteins deregulated at diagnosis and HIF-1α were included in the first analysis, and then networks were clustered to a specified number of clusters (n = 2), as follows: one cluster of proteins showing interactions with HIF-1α; and a second cluster of proteins with no known interactions with HIF-1α. A total of 47 proteins (39% of the included proteins, n = 120) clustered with HIF-1α and were mostly involved in the regulation of cellular metabolic processes and immune effector processes, such as CD4^+^ αβ T cell activation, Th1 and Th17 differentiation, and leukocyte activation (Figure 4A). Similarly, a total of 13 proteins (41% of the included proteins, n = 32) of those genes downregulated after alemtuzumab in responders clustered with HIF-1α (*AKT3*, *CDKN1B*, *FOXO1*, *KLF2*, *LEF1*, *MAML2*, *RUNX2*, *SATB1*, *SESN1*, *SESN3*, *STAT3*, *TXNIP*, and *WNT7A*) and were involved in metabolic processes, the regulation of oxidative stress, the regulation of TOR and mTORC1 signaling, and T cell differentiation (Figure 4B). Moreover, there were 77 pathways in common before and after treatment, while 59 (15.9%) were present only in responders after alemtuzumab and were mostly implicated in mTOR and mTORC1 regulation (Figure 4C). Therefore, even if we look at a different BMF syndrome (e.g., LGL or AA), we might discover recurrent involved proteins and pathways, confirming the common hypothesis that these syndromes share a similar pathogenesis that also translates in overlapping symptoms and manifestations.

## 6. Conclusions

In conclusion, acquired BMF syndromes are considered immune-mediated diseases because hematological recovery after immunosuppressive therapies is the strongest indirect evidence of the pathogenic role of immune cells, especially cytotoxic CD8^+^ T cells and Th1 cells. Type I interferons play an essential role in immune response polarization and the induction of cytotoxic activities against autologous HSPCs, and also exert direct pro-apoptotic and growth inhibitor effects. Historically, IFNγ and TNF-α have been implicated in AA pathogenesis. IFNγ produced by exogenous and stromal cells inhibits HSPC growth and reduces HSC self-renewal possibly by impairing TPO and other growth factor signaling pathways, likely acting as a decoy receptor. Furthermore, IFNγ directly suppresses erythropoiesis by blocking HPSCs in early stages of differentiation. Currently used immunosuppressive therapies could exert their clinical efficacy by directly blocking T cell differentiation and by indirectly interfering with type I IFN responses. The efficacy of TPO receptor agonist eltrombopag could be related to: (i) a direct stimulation of HSC growth; (ii) indirect immunomodulatory effects; and (iii) a decoy IFN receptor function. Moreover, JAK1/2 inhibitors are a promising therapeutic approach, due to their immunomodulatory and anti-inflammatory effects. Therefore, IFNγ still remains the central cytokine driver in acquired BMF syndrome pathogenesis, and an optimal therapeutic target using agents that directly or indirectly interfere with related signaling pathways.

## Data Availability

Data are available upon request by the authors.
